# Case Report: Successful treatment of drug-resistant epilepsy with unilateral deep brain stimulation of the anterior nucleus of the thalamus

**DOI:** 10.3389/fnins.2026.1978183

**Published:** 2026-10-19

**Authors:** Ryota Sasaki, Kengo Yamada, Kentaro Tamura, Masako Kinoshita, Young-Soo Park, Abbas F. Sadikot, Ichiro Nakagawa

**Affiliations:** 1 Department of Neurosurgery, Nara Medical University, Kashihara, Japan; 2 Department of Neurosurgery, National Hospital Organization Nara Medical Center, Nara, Japan; 3 Department of Neurology and Neurosurgery, Montreal Neurological Institute, McGill University, Montreal, QC, Canada; 4 Department of Neurology, National Hospital Organization Wakayama Hospital, Hidaka, Japan

**Keywords:** anterior nucleus of the thalamus, deep brain stimulation (DBS), drug-resistant epilepsy (DRE), epilepsy, focal epilepsy, neuromodulation, palliative surgery, unilateral stimulation

## Abstract

**Background:**

Deep brain stimulation of the anterior nucleus of the thalamus (ANT-DBS) is an established palliative treatment for drug-resistant epilepsy (DRE) and is conventionally performed bilaterally. However, the efficacy of unilateral ANT-DBS remains unclear. We report a patient with DRE who underwent unilateral ANT-DBS because of an epileptogenic network predominantly involving one side and anatomical constraints precluding electrode implantation on the contralateral side.

**Methods:**

A 29-year-old female underwent comprehensive presurgical evaluation, including seizure semiology assessment, video-electroencephalography (EEG), computed tomography, and magnetic resonance imaging. She developed DRE following multimodal treatment for a suprasellar germ cell tumor during childhood. Despite multiple antiseizure medications and vagus nerve stimulation, she continued to experience approximately 10 habitual seizures per day.

**Results:**

Seizure semiology was characterized by asymmetric tonic posturing of the limbs lasting 10–30 s, followed by an atonic phase. EEG findings indicated a widespread epileptogenic network predominantly involving the right frontal and temporal areas. Brain imaging showed severe left hemispheric atrophy, marked widening of the left subdural space, and a pre-existing shunt system, which prevented identification of the left ANT. Therefore, DBS implantation was performed in the right ANT alone. Stimulation was initiated 7 days after surgery at an intensity of 2 mA. Following initiation of right ANT stimulation, seizure frequency decreased from approximately 10 to one per day, likely due to a microlesioning effect. Thereafter, right ANT stimulation was associated with a sustained seizure reduction of approximately 60%. Postoperative EEG demonstrated a reduction in interictal epileptiform discharges. The clinical benefit was maintained at the 9-month follow-up, with no surgery- or stimulation-related adverse events were observed.

**Conclusion:**

Unilateral ANT-DBS can provide clinically meaningful seizure control in carefully selected patients whose predominant epileptogenic network is localized to one cerebral hemisphere. This approach may represent a feasible therapeutic option when anatomical or surgical constraints prevent conventional bilateral ANT electrode implantation. Further studies with larger patient cohorts and longer follow-up periods are required to clarify the indications, durability, and efficacy of unilateral ANT-DBS.

## 1 Introduction

Patients with drug-resistant epilepsy (DRE), whose seizures cannot be controlled by antiseizure medication (ASM) treatment alone, account for approximately 30% of all epilepsy patients (Kwan and Brodie, 2000). According to the recommendation by the International League Against Epilepsy, epilepsy surgery should be considered for DRE (Jehi et al., 2022).

Deep brain stimulation (DBS) is one of the palliative surgical treatments for DRE (Sasaki et al., 2026). The anterior nucleus of the thalamus (ANT) is the most common target. ANT-DBS is effective for focal epilepsy, with reports indicating a 75% reduction in seizures over a 10-year follow-up period (Salanova et al., 2021). In Japan, ANT-DBS for DRE was approved for insurance coverage in December 2023. As ANT-DBS typically employs bilateral ANT stimulation (Fisher et al., 2010; Salanova et al., 2021; Sasaki et al., 2026), the efficacy of unilateral ANT stimulation remains unclear. We report a patient who underwent unilateral ANT-DBS on the right due to an epileptogenic network predominant in the right hemisphere and anatomical limitations for electrode implantation on the left side, achieving significant improvement.

## 2 Case description

A 29-year-old woman with DRE underwent a detailed evaluation for surgical treatment. At age 7, she developed a suprasellar germ cell tumor and underwent craniotomy for tumor resection, high-dose chemotherapy, whole-brain radiation therapy with a local boost (50 Gy in total), and stereotactic body radiation therapy to the anterior spinal cord (24 Gy). The brain tumor went into remission, but she was left with panhypopituitarism and severe intellectual disability. The Wechsler Intelligence Scale for Children, Third Edition, at age 16 showed a verbal intelligence quotient (IQ) of 48, performance IQ of 47, and full-scale IQ of 42. A ventriculoperitoneal shunt was inserted for secondary hydrocephalus, but it caused intracranial hemorrhage due to overshunting and has been ligated since then.

At age 22, she experienced nonconvulsive status epilepticus associated with hyponatremia (Na 123 mmol/L, reference range 135–145 mmol/L), and brain magnetic resonance imaging (MRI) showed abnormal lesions in the bilateral temporal lobes. This was followed by recurrent epileptic seizures. Her family history was unremarkable. Levetiracetam and carbamazepine were ineffective, leading to referral to our epilepsy clinic. Add-on therapy with perampanel and lacosamide caused side effects and was not tolerated. Vagus nerve stimulation (VNS) was implanted at age 23, which reduced seizures with abrupt falls from several times to once or twice per week, but daily seizures could not be controlled. Other ASMs, including valproic acid, topiramate, and brivaracetam, were tried but had limited effects. At age 29, the patient underwent unilateral ANT-DBS treatment after a comprehensive assessment for further surgical treatment, which successfully suppressed her seizures.

In this study, we thoroughly investigated her seizure semiology, electroencephalogram (EEG) and brain imaging findings, and clinical course to characterize the clinical features associated with a favorable response to unilateral ANT-DBS.

This study was conducted in accordance with the principles of the Declaration of Helsinki, and the case report was prepared in compliance with the CARE guidelines. Approval of the study design was waived by the Ethics Committee of Nara Medical University based on its classification as a retrospective analysis of anonymized clinical data. Written informed consent was obtained from the patient.

## 3 Diagnostic assessment

Neurological examination revealed right-sided hemiparesis with a manual muscle test grade of 3. She produced no meaningful speech and was unable to follow verbal commands. Neuropsychological assessment could not be performed. The seizure semiology comprised asymmetric tonic posturing lasting 10–30 s, characterized by eye deviation to the left, extension of the left upper limb, and flexion of the right upper limb. Following the tonic phase, all four limbs became flaccid and the trunk tilted to the left side.

Figure 1 and Supplementary Figure 1 show brain imaging findings. Head computed tomography (CT) showed brain atrophy, hydrocephalus, calcifications in both basal ganglia, and post-craniotomy changes in the right frontal region. Brain MRI revealed post-tumor resection brain damage with marked atrophy in the left hemisphere. The left ANT could not be identified.

**FIGURE 1:**
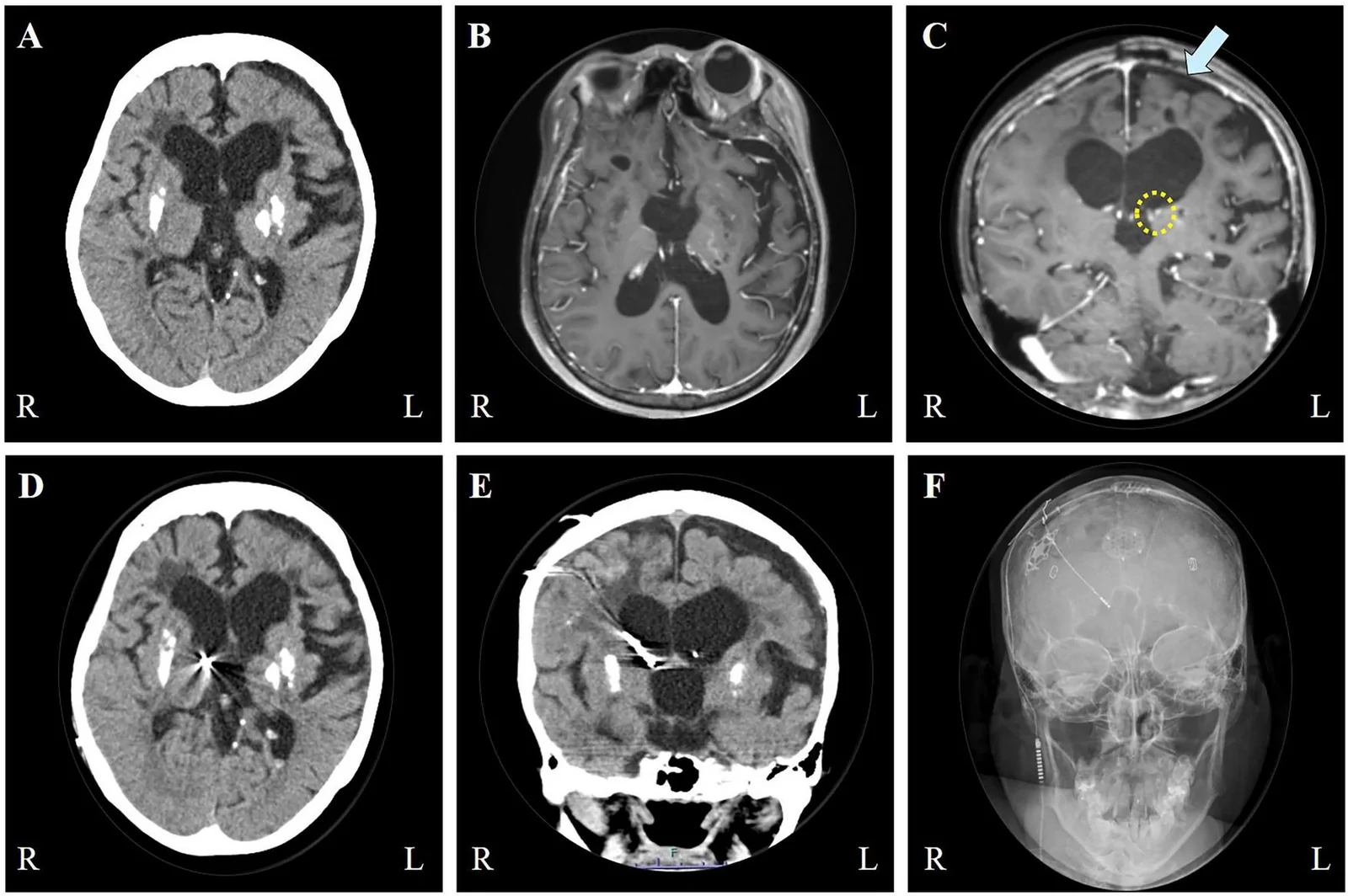
**(A)** Preoperative head computed tomography (CT) demonstrated cerebral atrophy, hydrocephalus, calcification of the basal ganglia, and postoperative changes related to the previous right frontal craniotomy. **(B,C)** Preoperative brain magnetic resonance imaging also showed postoperative changes following brain tumor surgery. Cerebral atrophy was particularly severe in the left hemisphere, with marked widening of the subdural space (light-blue arrow), and the left anterior nucleus of the thalamus (ANT) could not be identified (yellow dotted circle). **(D,E)** Postoperative head CT and **(F)** skull radiographic image confirmed placement of the deep brain stimulation electrode in the right ANT.

Video-EEG was performed for 3 days using EEG-1200 (Nihon-Kohden, Tokyo, Japan) via scalp electrodes placed according to the International 10–20 system with T1/T2. Interictal awake recording revealed a disorganized posterior dominant rhythm of 6–7 Hz and frequent slow waves and sharp waves in F4 and C4, with frequent spikes and high-amplitude fast waves in the same areas occurring at sleep onset (Figure 2A). She slept for approximately 8 h per day. During sleep, EEG showed disrupted sleep architecture without obvious slow-wave sleep, intermixed with abnormal theta and delta activities maximal in the left frontal region. Independent interictal epileptiform discharges (IEDs) were also observed in the right frontotemporal region. Habitual seizures were recorded approximately 10 times per day. Ictal EEG showed a high-amplitude burst of spiky fast waves originating from F4 and spreading to the right hemisphere (Figure 2B). Frequent subclinical EEG seizures were observed during sleep.

**FIGURE 2:**
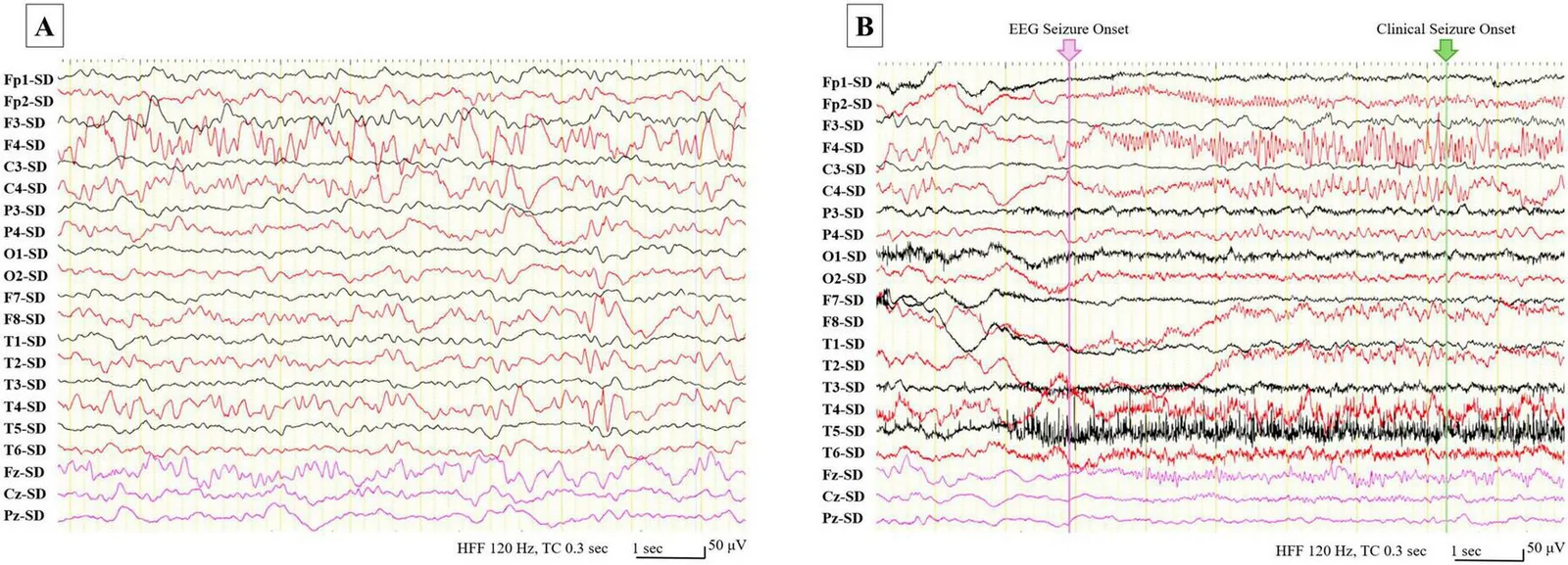
**(A)** Preoperative interictal electroencephalography (EEG) demonstrated a disorganized posterior dominant rhythm at 6–7 Hz, with frequent slow waves and sharp waves over the F4 and C4 regions. During sleep, frequent spikes and high-amplitude fast activity were observed over the F4–C4 region. Independent interictal epileptiform discharges were also recorded from the right frontotemporal region. **(B)** EEG recorded during a habitual seizure. The electrographic seizure arose from F4 and spread throughout the right hemisphere (pink line). Clinical seizure onset occurred approximately 5 s after the electrographic onset (green line). High-frequency filter, 120 Hz; time constant, 0.3 s.

Based on these findings, the patient was diagnosed with focal epilepsy arising from the right frontal and temporal lobes. Although the seizure semiology was identical, focal resection was not indicated because of the presence of a widespread epileptogenic network. Therefore, ANT-DBS was considered. Initially, conventional bilateral ANT electrode placement was planned; however, we could not identify the left ANT (Supplementary Figure 1; Amunts et al., 2013). Moreover, severe atrophy of the left hemisphere, resulting in an excessively widened subdural space, and a shunt valve located at the anticipated burr-hole site, with thinning of the overlying scalp, hindered appropriate electrode placement in the left ANT. Additionally, in this case, the epileptiform abnormalities were predominantly localized to the right frontal region. Previous animal DBS studies and case reports of responsive neurostimulation (RNS) in humans demonstrated seizure reduction with unilateral ANT stimulation (Elder et al., 2019; Hamani et al., 2004; Herlopian et al., 2019; Zhang et al., 2012). The proposed surgical plan was explained in writing to the patient’s legally authorized representative, and written informed consent was obtained. Consequently, ANT-DBS was performed on the right side only (Figure 1).

Stimulation commenced 7 days postoperatively at an intensity of 2 mA. Around this time, epileptic seizures markedly decreased to approximately once per day, which was considered to reflect a microlesioning effect rather than the effect of stimulation itself. Thereafter, the seizure reduction rate stabilized at approximately 60% and was maintained throughout the 9-month postoperative follow-up period (Figure 3). ASMs were continued unchanged after surgery. The duration of seizures was also reduced by approximately half, resulting in fewer interruptions of daily activities. In addition, improved daytime alertness, which may suggest an improvement in nighttime sleep quality contributed to an improvement in the caregiver’s quality of life. Postoperative routine 30-min EEG during an awake demonstrated a reduction in IEDs (Figure 4). The effect persisted at the 9-month postoperative follow-up. DBS parameters were adjusted to 2 mA, 145 pulses/s, 90 μs, and 1 min on/5 min off. VNS stimulation intensity was reduced to 1 mA (30 Hz, 250 μs, 30 s on/1.8 min off). No stimulation-related complications were observed.

**FIGURE 3:**
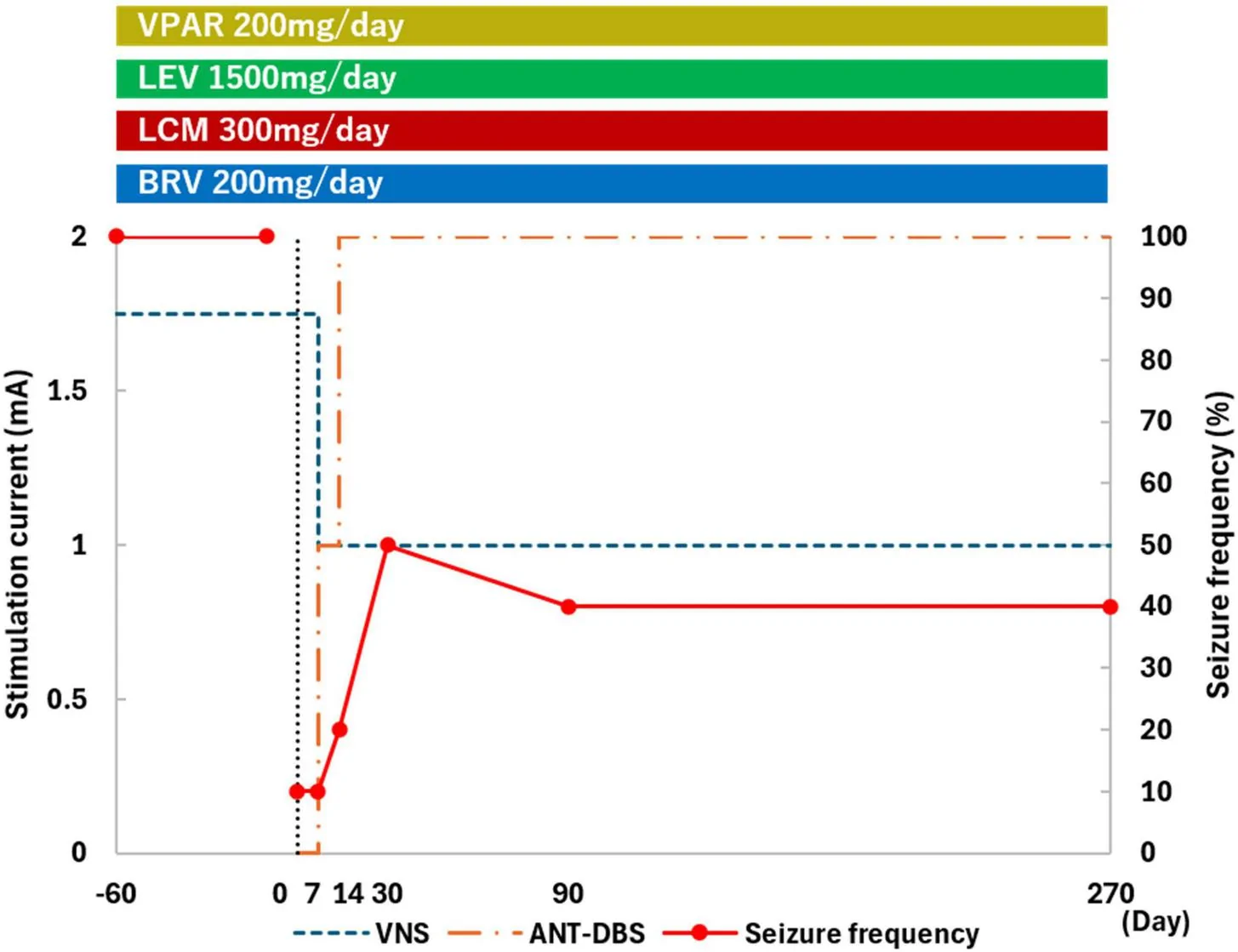
The upper bar graph shows the dosage of antiseizure medications. The lower line graph shows changes in seizure frequency (red line) and amplitude of vagus nerve stimulation (VNS) (blue line) and deep brain stimulation of the anterior nucleus of the thalamus (ANT-DBS) (orange line). Seizure frequency is expressed as a percentage of the preoperative baseline, which was defined as 100%. The vertical dotted black line indicates the day of ANT-DBS implantation. BRV, brivaracetam; LCM, lacosamide; LEV, levetiracetam; VPA, valproic acid.

**FIGURE 4:**
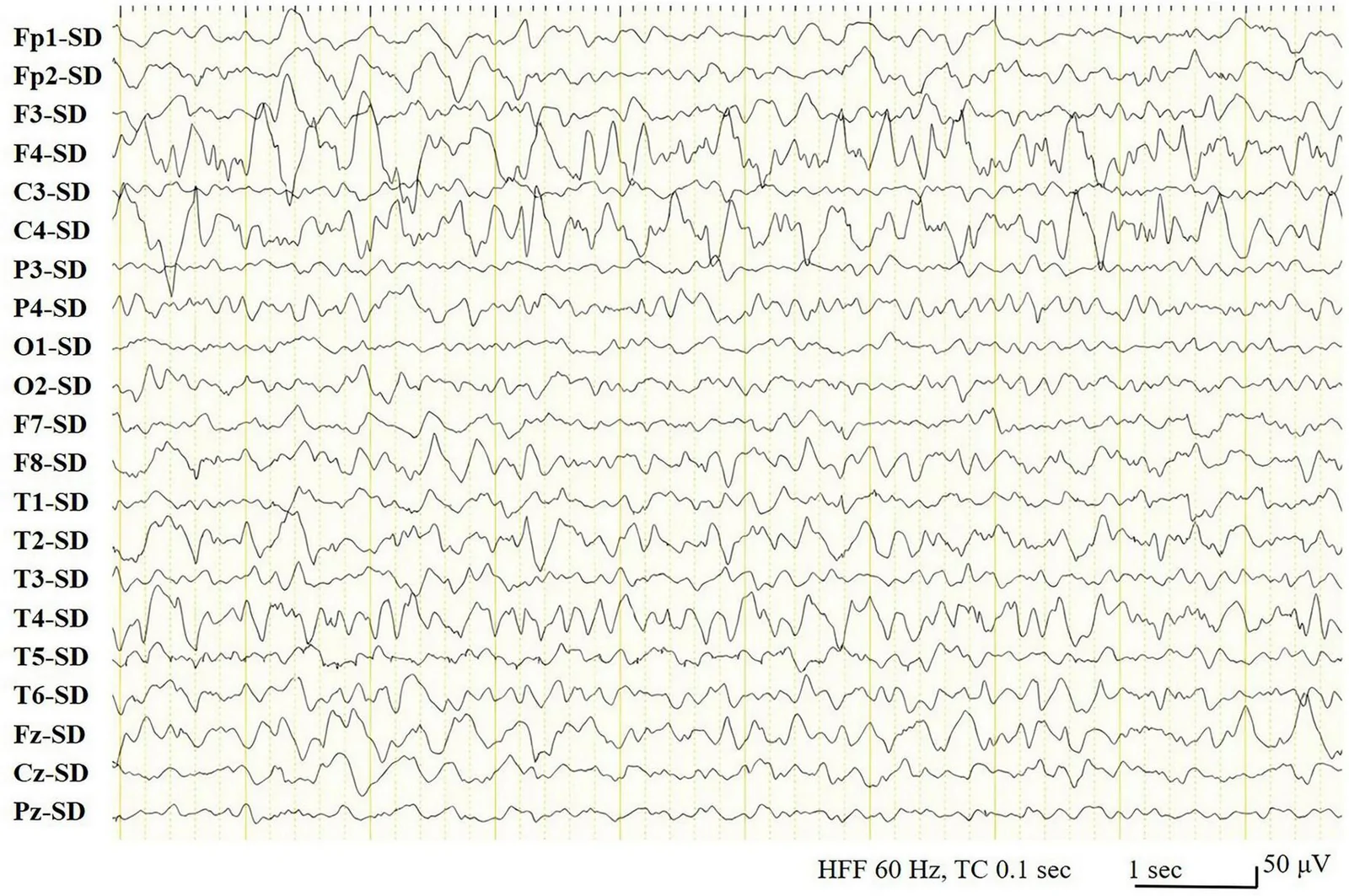
Postoperative interictal electroencephalography following right anterior nucleus of the thalamus deep brain stimulation demonstrated a reduction in interictal epileptiform discharges. High-frequency filter, 60 Hz; time constant, 0.1 s.

## 4 Discussion

In this case, owing to the localization of the primary epileptogenic network and anatomical constraints, DBS was performed in the right ANT only. Postoperatively, sustained seizure control was achieved without any notable adverse events. Recently, Mitsuhashi et al. (2026) reported a case in which, after right frontal disconnection and right temporal lobectomy, unilateral left ANT-DBS was effective for drug-resistant epileptic spasms. We were able to further evaluate the direct seizure-suppressive effect of unilateral ANT-DBS in a patient who had not undergone resective epilepsy surgery. In addition, we observed a reduction in seizures characterized by bilateral asymmetric tonic posturing.

Deep brain stimulation provides a reversible and adjustable modulation of brain function, and bilateral DBS is generally recommended for the treatment of movement disorders that commonly produce bilateral symptoms, including essential tremor, Parkinson’s disease (PD), and generalized dystonia (Kumar et al., 1999; Ondo et al., 2001; Vidailhet et al., 2007). In a recent systematic review, unilateral DBS targeting either the subthalamic nucleus (STN) or globus pallidus internus (GPi) in patients with PD improved the Unified Parkinson’s Disease Rating Scale Part III scores by 37%, whereas bilateral DBS resulted in improvements of up to 66% (Vetkas et al., 2025). Unilateral DBS improved contralateral symptoms by up to 75% but produced only 28% improvement in ipsilateral symptoms (Vetkas et al., 2025). Nevertheless, unilateral DBS may be considered when symptoms are markedly asymmetric, disabling symptoms are largely confined to one side of the body, and contralateral symptoms are mild and do not require immediate treatment (Slowinski et al., 2007; Vetkas et al., 2025). In such cases, a staged surgical strategy, with subsequent conversion to bilateral DBS if the disease progresses, may also be considered (Golubovsky et al., 2020). In summary, unilateral DBS is primarily effective for symptoms mediated by the ipsilateral hemisphere. Therefore, bilateral DBS is preferable when therapeutic effects involving both cerebral hemispheres are required.

One rationale for performing bilateral DBS in patients with DRE is that, even in focal epilepsy, seizures may not be confined to the cortex or basal ganglia of a single hemisphere. Rather, they can readily propagate through the thalamus to widespread ipsilateral and contralateral cortical networks. Accordingly, modulation of the bilateral networks involved in seizure propagation is often considered necessary. When a clearly localized, unilateral, single epileptogenic focus is identified, resective surgery should be considered, as it can achieve seizure freedom in approximately two-thirds of patients with medial temporal lobe epilepsy and about half of those with neocortical epilepsy (Engel et al., 2003). The therapeutic effect of unilateral ANT-DBS for DRE has not been fully investigated in humans. A rat study with unilateral amygdala kindling reported that high-frequency stimulation of the ipsilateral ANT resulted in a significant reduction in seizure frequency and duration; in contrast, stimulation of the contralateral ANT did not yield a significant seizure suppression effect (Zhang et al., 2012). Furthermore, in a rat model of secondary generalized seizures induced by intraperitoneal pilocarpine injection, significant seizure suppression was achieved only with bilateral ANT stimulation or destruction, whereas unilateral ANT stimulation alone did not suppress seizures (Hamani et al., 2004). Although there are efferent fibers from the ANT that project bilaterally, unilateral ANT stimulation alone likely fails to produce sufficient therapeutic effects extending to the contralateral side (Shibata, 1993).

Bilateral asymmetric tonic posturing with preserved consciousness at seizure onset in our patient can be associated with focal seizures arising from the supplementary motor area (SMA; Alonso-Vanegas et al., 2017; Bagla and Skidmore, 2011; Sitthinamsuwan et al., 2016). This semiology is thought to reflect seizure onset within the unilateral SMA, because tonic posturing occurs at seizure onset and the small size of the SMA allows rapid propagation of epileptic activity, resulting in widespread or generalized motor manifestations (Buonocore et al., 2026; Ohara et al., 2004). However, the subsequent prolonged atonic phase and impaired consciousness differed from the seizure semiology typically observed in unilateral SMA seizures. These findings suggested the presence of an epileptogenic network involving both cerebral hemispheres. In particular, the predominant epileptogenic network was distributed in the right frontal region, and seizure control was achieved with stimulation of the right ANT alone. These findings are therefore consistent with the observations described above.

Several case reports described favorable seizure control achieved with unilateral ANT stimulation using responsive neurostimulation (RNS). Herlopian et al. (2019) implanted RNS in the bilateral posterior frontal cortex and ANT in a patient with refractory genetic generalized epilepsy. By stimulating the right ANT and prefrontal cortex, which were contralateral to the dominant side of the seizure symptoms, they achieved a 90%–95% reduction in seizures over 2 years (Herlopian et al., 2019). Elder et al. (2019) examined three cases of drug-resistant multifocal epilepsy primarily treated with unilateral ANT-RNS, confirming a 50%–56% seizure improvement during 33–35 months of follow-up. While the mechanisms of closed-loop stimulation strictly differ from those of ANT-DBS, these studies clearly demonstrate the importance of targeting the hub of the epileptogenic network. In the current study, stimulation of the ANT alone also achieved favorable seizure control, further supporting this hypothesis. Moreover, unlike RNS, DBS delivers ongoing stimulation during the interictal period, and its therapeutic effects may therefore evolve over the long term.

The mechanism by which ANT-DBS suppresses epileptic seizures is not fully understood. However, its particular effectiveness in temporal lobe epilepsy may be related to the ANT and medial temporal lobe region being components of the limbic system (Fisher et al., 2010). Although not exclusive to ANT-DBS, previous studies have reported a stronger functional connectivity between the thalamus and the seizure onset zone (Damiani et al., 2025; Xu et al., 2023). The present case study suggests that seizure suppression achieved by stimulating a unilateral ANT can be particularly effective for focal seizures involving the epileptogenic network of the ipsilateral hemisphere. This implies that in cases of bilateral foci, such as post-encephalitic epilepsy, bilateral ANT stimulation is necessary for achieving therapeutic effects (Sasaki et al., 2025). In our patient, unilateral ANT stimulation yielded a relatively favorable therapeutic response, suggesting that an etiology such as encephalitis was unlikely to have contributed substantially to the epileptogenic network.

This case study has several limitations. First, the follow-up period was relatively short, and long-term outcomes could not be assessed. Second, the patient had undergone VNS before ANT-DBS, and the extent to which VNS contributed to the observed therapeutic effect remains unclear. A synergistic effect between DBS and VNS may also have contributed to the favorable clinical course. Further accumulation of cases and additional studies are required to address these concerns.

## 5 Conclusion

When the epileptic focus is localized to one cerebral hemisphere, unilateral ANT-DBS alone may achieve favorable seizure control. Attempting unilateral ANT-DBS is particularly worthwhile when anatomical limitations prevent electrode placement in both ANTs.

## Data Availability

The raw data supporting the conclusions of this article will be made available by the authors, without undue reservation.

## Supplementary material

Supplementary Figure 1 Comparison of the preoperative contrast-enhanced coronal T1-weighted brain MRI **(A)** with the anterior nucleus of the thalamus (ANT) delineated on 200-μm sections from the BigBrain Project **(B)** (Amunts et al., 2013). **(C)** Shows an enlarged view of the area outlined by the green dotted line in panel **A**, whereas **(D)** shows an enlarged view of the area outlined by the red dotted line in panel **B**. The ANT is indicated by the yellow ovals. The right ANT was identifiable, whereas the left ANT could not be identified because of severe left hemispheric atrophy.
